# Diphtheria with Cases

**Published:** 1899-04

**Authors:** S. A. Kimball

**Affiliations:** Boston, Mass.


### DIPHTHERIA WITH CASES.

S. A. Kimball, M. D., Boston, Mass.

There is probably no disease so feared by physicians as this. Case after case will yield readily to remedies, and then there will come a series which will be lost in spite of every endeavor.

Some of these would undoubtedly be saved if we possessed the requisite knowledge of the materia medica and the skill to properly apply it. But there are others in which the onslaught of the disease is so sudden and overwhelming that there is no time to resist, and nothing seems to help.

Of course, in these latter, as in all cases, there is a deeply seated psoric miasm, to which the disease sets fire, as it were, and the result is severe or otherwise in proportion to the resistance of the vital force.

In the treatment of such conditions the remedy is of first importance, then plenty of fresh air, sunshine, and proper nourishment.

Cases will recover under the indicated remedy in most unfavorable surroundings, but the best of hygienic conditions will not save a critical case that is not prescribed for accurately. So that good nursing and surroundings, with the correct prescribing are all essential in obtaining the most satisfactory results. Fresh air and sunshine are of the utmost value, and are the best disinfectants.

No odorous or non-odorous mixtures under that name should be allowed in any sick room, with the possible exception of charcoal.

They substitute one smell for another, and in either case pollute the air which our patient is expected to breathe. The day of stuffing sick people with food, whether they wish it or not, seems to be waning, fortunately for the patient and for us, also.

It is nearly always safe to allow patients to eat when they are hungry, and not otherwise.

Hunger is the best indication for the need of nourishment, and if food is given without this craving of the system it will act as an irritant to the stomach and bowels.

It is not the amount taken but the amount absorbed that is important. Diphtheria, however, may be an exception to trusting to a patient's sense of hunger, for in cases where the pain on swallowing is severe, many of them, especially children, will prefer to be hungry rather than to suffer the agony of swallowing. It is often necessary, therefore, to insist upon the taking of nourishment at stated intervals.

Liquids, being usually most easily swallowed, are chiefly indicated. Meat broths, made with rice or other vegetables, milk, gruels, fruit juices, ice cream, in fact about everything in reason that the patient can swallow. No alcoholic stimulants should be given, for as action and reaction are equal and opposite, all stimulation from alcohol is followed by just as much reaction, and with the lowered nervous vitality in diphtheria and consequent weakness of the heart an alcoholic stimulant is a dangerous expedient.

In prescribing for the symptoms that make up a case of diphtheria, it is necessary to use the utmost care, for there may not be time to rectify a wrong selection. The general symptoms are of first importance, those which affect the patient as a whole without regard to the condition of his throat; then the mental symptoms, if there are any, and then the particular symptoms of the throat itself. Often we can obtain no other symptoms but the local manifestations, and then it is important to know where the membrane first appeared, and in which direction it extended; external sensitiveness of the neck; swelling of the glands; aggravation or amelioration from hot or cold drinks, from liquids or solids: extension of the pain on swallowing to the ears, or other parts; appearance of the membrane, etc. All of these are of the utmost importance, and often are all we have to guide us in the selection.

Of course, it is not necessary here to speak of the harmful effects from the use of gargles or topical application. They not only weaken the patient, but distort or destroy valuable indications for the remedy.

We have all had plenty of Lachesis and Lycopodium cases in which the membrane has melted away in twenty-four, thirty-six, or forty-eight hours, and the patient has speedily recovered, but I have one or two cases here in which they have not recovered, and had I known my materia medica better and been better able to apply it, the results, I think, would have been very different.

It is remarkable to what extent the mucous membrane can be involved without the patient being seriously ill. I recall the case of a child three years old, in whom the membrane, beginning in the throat, extended to the nose, and then to the larynx, and yet he had to be held most of the time to prevent his getting down on to the floor to play.

In contradistinction to this the following fatal case is given, which occurred in the second year of my practice. The patient was a boy eight years of age, and when I first saw him there was a dirty green patch of membrane on the left tonsil, with soreness of the left side of the neck externally. He received one dose dry of Lach., CM. (Swan).

The next day the throat was about the same, but he felt better generally.

The third day a patch of white membrane appeared on the right tonsil, but both this membrane and that on the left tonsil appeared loose and cheesy, and as he was generally feeling better no remedy was given.

The fourth day the throat was better, but he complained much of aching in his back and limbs, with great restlessness.

Here is where I made a "fatal error," and prescribed a dose of Rhus., CM. (Swan).

The next day there was more membrane on the right tonsil, involving the right side of the uvula, but the left tonsil was nearly clean.

During the night he had been very restless with a throbbing headache, jerking and twitching of muscles, and moaning in sleep, which conditions were now present with flushed face and hot head.

He received a dose of Bell., CM. (Swan).

That night he had an occasional croupy cough, and the next day the cough being much worse and croupy, he was given a dose of Kali-bich., CM. (Swan).

The cough was better for a day, then became worse and more croupy, with nose bleed, and he was given Kali-bich. in water, several doses.

That evening the late Dr. Ballard, who was visiting relatives in the town, saw him with me, and advised Lyc. in water, a few doses.

He seemed slightly better that night, but in the morning became comatose and died at 10 A. M.

It was Dr. Ballard's opinion, and is now mine, that if I had not interfered with the action of the Lach. by giving the dose of Rhus. he would have had a much better chance for recovery, and, in fact, it is doubtful if the disease would have at all increased had I known enough to let him alone.

The following cases occurred in the winter of 1895-96:

There were two children in the family, three and five years of age, and both had been having an attack of whooping cough, from which they had apparently recovered.

December 1st, 1895, the elder, a girl, had a left-sided diphtheria, which extended to the nose.

She also coughed up pieces of membrane, but there seemed to be no extension to the larynx.

There was also a small patch of membrane on the inner right side of the mouth. A dose of Lach., 1M. (Fincke) followed by a dose of Lyc., CM., and later a dose of Lac-can.,

CM., relieved her so that by December 10th she was apparently well.

December 12th her little brother, three years old, was delirious in the night, and the next day a membrane appeared on both tonsils, worse on the right.

There was much swelling of the glands of the neck, externally, and later the membrane extended to the larynx with loss of voice, croupy cough, and stringy expectoration.

Two or three remedies had been given as they appeared indicated without much apparent effect, but for this latter condition Kali-bich. 200 was given in water for several doses, which seemed to clear up the whole condition, so that by January 1st his voice had returned, his throat was clear, and he seemed well.

I did not see him again for two weeks, when I was called becaused he complained of aching in the nape of the neck.

There was weakness of the muscles of the neck, weakness oi the knees, tottering gait, and evidences of post-diphtheretic paralysis. He received a dose of Causticum.

When a week later, his condition being about the same, he began to have spasmodic attacks of wheezing, choking cough, with whooping as if the former whooping cough were returning, but it was not the same as before, and from the description was worse than anything he had ever had.

After waiting several days for it to pass away, which it did not do but continued to grow worse, he was given a dose of Spongia. The attacks in a day or two were less frequent and less violent, and everything appeared to be moving in the right direction. The fourth day after the Spongia he was taken suddenly in the night with a convulsion and was dead when I reached the house. He had complained of nothing in particular during the day except that he felt tired.

Now this is a case in which it would seem that if the correct remedy had been given the boy would have lived, and yet the apparently indicated remedies seemed to have a favorable effect.

The action of Causticum was not interfered with until the attacks of choking and wheezing became so alarming that it seemed as if he would not come out from them. There was no sudden cessation from the Spongia but a gradual improvement as would be expected from a properly selected remedy, and the convulsion was as unexpected as a thunderbolt from a clear sky.

The following case was cured with Merc-iod. flavus:

A boy eight years old, both tonsils swollen, with patches of membrane, worse on the right side.

Aggravation from cool drinks; dazed on waking; cries and afraid on waking, and does not seem to know where he is. Generally worse in the afternoon. Lyc. in two different potencies had seemingly no effect.

The fourth day there were the following conditions: Large yellow patch on right tonsil; tongue thickly coated, yellowish white; uvula elongated; aggravation from cold drinks.

One dose, dry, of Merc-iod. flavus, CM. (F.) cleared everything up in two days.

### Discussion.

Dr. Kennedy—I would like to ask the doctor if he found "the bug" in these cases?

Dr. Kimball—In some of them; others seemed to be bugless.

Dr. Pease—In view of these cases of that terrible disease, and also of the fact that what may appear to be an ordinary sore throat sometimes proves to be true diphtheria, are we not remiss in our duty if we do not study carefully what seems to be even a simple case? I know several cases where serious cases of diphtheria have developed after two or three days of apparently ordinary, simple sore throat. We should very carefully work out that first prescription. We all remember Kent's article on "The First Prescription," printed some years ago.

Dr. Adams—It has been my experience that the more simple the case the more serious it will be apt to become.

Dr. Patch—I hope this paper of Dr. Kimball's will be thoroughly discussed. It is of particular interest to us. It is a subject always important owing to its seriousness. We must show what we can do with our remedies, whether we can do better work than the others. My experience has been so unfortunate with this disease that I may perhaps feel more strongly on that account. I have recently had three cases. Each of them was carefully studied, not only by myself, but with the aid of the best counsel I could get. We failed on each of them. The fourth was taken out of our hands, treated with Anti-toxin, and also died.

Dr. Kennedy—I have not had many cases of diphtheria, but in the earlier years of my practice I had a bitter experience with the disease. I had several cases that died. I did the very best I could with them, and I consulted older men, one of whom had been in practice some time. He told me to spray the throat with alcohol, which I did. I might as well have thrown it against the side of the house, as far as results were concerned. I am inclined to think that there is a certain class of cases of diphtheria that are doomed from the first, but that is no reason why we should not from the first give careful attention to the individual case before us, and as has been implied, avoid careless prescribing, thinking it is a case of ordinary sore throat. Those cases which proved to be fatal were characterized by great glandular swelling in the cervical and submaxillary glands, so that almost a deformed appearance was presented. The membrane was about the color of cast iron that had been polished, a dark gray color, thick and leathery, odor strong—so strong in those cases that my clothing was saturated; marked salivation; great prostration. The children were scrofulous.

The cases I have had later have been milder and characterized by different symptoms. There has been membraneous deposit, more or less prostration, foul breath, coated tongue, but not swollen glands. I will say that in a number of the cases which proved fatal the disease invaded the larynx. I got so after a little that when I had a case of diphtheria and the patient began to have that croupy sound, I began to wish I was somewhere else, because they usually died. There is one bright spot, however. I was called by a family where a little fellow had just died of diphtheria. His sister had been taken, and they wished different treatment for her. I studied her case. The disease invaded the larynx, and the croupy symptoms came on. She was about fifteen years old. I found Kali-bich. the remedy, and under it the membrane loosened, and quite a considerable piece was expectorated, which I preserved quite a number of years as a trophy. It was irregular in form, about one and one-half inches long and about one-half inch wide. The girl made a good recovery, and is living and well to-day. I want to emphasize the necessity of giving special attention to this disease. We have to meet people who believe in Anti-toxin, and it is considered almost criminal if it is not given. We need to study these cases thoroughly.

Dr. Pease—In connection with diphtheria I want to speak of one case in which a rather unusual remedy was used and a perfect cure resulting—that remedy is Cuprum-sulph. It was a child between three and four years of age. The diagnostic symptoms were all present. The child would put its hand up to the back of its neck when I tried to find on which side the pain was. The membrane was a dingy brown, and was here and there over the pharyngeal wall, on tonsils, uvula, and palate. Upon these symptoms I prescribed Nitric-acid, with no results at all. The second day I gave Lachesis, without benefit. A few weeks previous to this I had had a case of ulcerating vulva in a female patient with just such colored membrane all over the mucous surfaces of the labia and with similar discharge. This child's appearance reminded me so strongly of the conditions in that patient, that not seeing clear indications for other remedies, I ventured to give Cuprum-sulph., 50M. Clinching the hands and itching of the face were other symptoms which reminded me of Copper. On the evening of that day the first encouraging sign appeared. I watched the case carefully, treating with Sac-lac., and the case made a complete recovery. It is the only case of diphtheria in which I have used Cup-sulph. I am very much interested in the remedy, and have come to use it frequently, especially in cases of eroded, foul, exuding, painful vulvæ, with rank, profuse, brownish leucorrhœa. In one case particularly those brownish little patches much resembled a false membrane. The general characteristics of the remedy, so far as I have observed, are general fetor, ulceration, and profuse discharge. Zinc-sulph. is similar, especially in its action on the genitalia.

Dr. Close—I agree with Dr. Kennedy that there is a certain class of cases of diphtheria which are doomed from the beginning, and happy is the man who does not meet them. I have been very fortunate in only meeting three such cases. The first one was an infant nine months of age, in whom no symptoms were noticed by the parents until it had entered into a comatose condition, when I was called and found it impossible to help. It was a case of sheer neglect, although it might be difficult to detect symptoms in a child of that age. The second case occurred under the most discouraging circumstances. A girl fourteen years of age, the worst deformed little creature I ever saw. She had been a poor, patient little sufferer all her life, and had a preternaturally sweet spirit. The pathos of such a life is inexpressible. She lived nine days and died, or died nine days and lived! The third case was that of a young woman attending school, who was of a very reticent, reserved disposition, and morbidly sensitive. She came home from school one day, said nothing, but laid down on her bed and went to sleep, her mother said. In the evening her mother sent for me and told me her daughter would not wake up. I examined her and found she was comatose, with a horrible fetor from the breath, which pervaded the room, as if decomposition had already set in. Blue hands and feet. She did not regain consciousness, and died during the night.

The remainder of my cases have been mostly mild, although I have had some severe cases with glandular involvement, and two cases of membranes in the larnyx and bronchial tubes. In both these cases there was a long struggle before recovery took place. I can give no special indications now, but the remedies which have been most serviceable to me have been Lachesis, Lac-can, Lycop., and Kali-bich. The mercuries I have never used with any degree of satisfaction. Have prescribed the Iodides of Mercury, but without good results.

Dr. Kimball—I did not speak of Anti-toxin in my paper, hoping that it would be brought out in discussion. As Dr. Kennedy said, we have it in our section to combat with. Results with this are so much more favorable than the former results of old school treatment that it seems to the people that Anti-toxin cures all cases. It undoubtedly cures some cases. It must be remembered, however, that Carbolic-acid is used in most of the preparations, and if you have read provings of Carbolic-acid, you will see why it may cure some cases. In an epidemic about three years ago in a neighboring town where homœopathic practice was good, bad, and indifferent, the percentages of cures by homœopathists was much in excess of those using Anti-toxin.

Dr. Morgan—I once had in one family seven patients with diphtheria, four of whom died. There were peculiarities in those cases which I think I ought to mention here. In the first place the membrane appeared on the tonsils, and at the sides, and under the strips of membrane ulceration extended into the submucous tissue. The membrane was rather a canary yellow. In some cases it would spread extensively without ulceration. It would extend up into the nasal cavity, forward into the soft palate, downward into the fauces and larynx. There was no external swelling, but there was a great deal of soreness and exceedingly bad odor. Swallowing was very much interfered with by the soreness. I called to my assistance several physicians. But they could do no good. They all agreed on using Bromo-chloride as a disinfectant. There was great alarm about contagion, and nobody could find where the disease came from. In cases of severe poisoning the breath of a patient would smell like an old filthy cesspool. Arsenicum was used, but it seemed to do no good. Crotalus and Kali-bich. seemed to do some good. Lac-can. helped some cases. Another peculiarity about these cases was that about two days before they died several little red-pointed dots appeared about the lips. Four of the patients died. The father and mother and next to the youngest child got well. Then I made special inquiry if anybody had taken the disease from them, but none could be found. I requested the plumbers to come and examine the waste pipes from the water-closet. They found below the funnel a small opening where considerable air came through, which did not smell, but the pipe went down and back into the yard and emptied into a cesspool. On opening the cesspool there appeared to be the same odor that was in the breath of the patients before they died. The children who were first taken had their room near where this first opening was, and the hall went forward into the house where the parents and the baby had been sleeping. I could find no other source for the disease than the cesspool. Afterwards I found that another family had lived in the house and left it because it was unhealthy. About three months after that I met with three other cases in another family in a different part of the town. Good sanitary conditions but much the same condition of membranes. I commenced to treat them. I treated two patients lying in the same bed, and saw not one bit of effect of any remedy I gave. They were using disinfectants—chlorides. I was in great distress, because I did not want to lose those children. Then it came into my mind that if chlorides could kill microbes, which might be treated with Sulphuric-acid and live afterwards, they might also kill children. I immediately told the mother to take the chlorides out of the house. I opened the windows and doors and directed that a dose of the same remedy, which I think was Kali-bich., be given to them every hour until I came back. When I returned I found improvement. I continued the treatment and the children got well. I have only lost one case since then, and I have had other cases of diphtheria of very disagreeable nature.

